# Nasal nebulization inhalation of budesonide for chronic rhinosinusitis with nasal polyps: A protocol for systematic review and meta-analysis

**DOI:** 10.1097/MD.0000000000020354

**Published:** 2020-05-29

**Authors:** Peng-ju Zheng, Yi-ying Zhang, Shu-hua Zhang, Gui-fang Liu, Jin-sheng Wang

**Affiliations:** a Department of Otolaryngology, First Affiliated Hospital of Jiamusi University; b Department of Otolaryngology, Jiamusi University Affiliated Stomatological Hospital; c Department of Otorhinolaryngology, Second Hospital of Jiamusi Agricultural Reclamation, Jiamusi, China.

**Keywords:** budesonide, chronic rhinosinusitis, efficacy, nasal nebulization inhalation, nasal polyps, safety

## Abstract

**Background:**

Chronic rhinosinusitis and nasal polyps (CRNP) is a common public health concern for general population, and is thought to negatively impact their quality of life. Although previous studies have reported that nasal nebulization inhalation of budesonide (NNIB) can benefit patients with such condition, its conclusions are still inconsistent. Thus, this study will assess the efficacy and safety of NNIB for the treatment of CRNP.

**Methods:**

To identify any associated studies, we will comprehensively and systematically search Cochrane Library, PubMed, EMBASE, Web of Science, PsycINFO, Cumulative Index to Nursing and Allied Health Literature, Allied and Complementary Medicine Database, Chinese Biomedical Literature Database, and China National Knowledge Infrastructure. We will search all electronic databases from inception to the present with no limitations of language and publication status. Two independent reviewers will undertake selection of study, data collection, and study quality evaluation, respectively. Another reviewer will help to settle down any different opinions between both of them. Study quality will be checked using Cochrane risk of bias tool, and statistical analysis will be performed using RevMan 5.3 software.

**Results:**

This study will assess the efficacy and safety of NNIB for the treatment of CRNP through assessing primary outcomes of nasal symptoms and polyp sizes, and secondary outcomes of serum cortisol levels, health-related quality of life, and any expected and unexpected adverse events.

**Conclusion:**

The results of this study will summarize the up-to-date evidence on assessing the efficacy and safety of NNIB for the treatment of CRNP.

**Study registration number:**

INPLASY202040108.

## 1 Introduction

Chronic rhinosinusitis and nasal polyps (CRNP) is one of the most severe of chronic rhinosinusitis.^[1–5]^ It is characterized by eosinophilic inflammation, and it manifests as nasal and facial congestion, loss of smell sense, rhinorrhea, and post-nasal drip.^[6–9]^ Epidemiologic studies reported that the prevalence rates are variances in different countries with 13% (USA), 11% (Europe), 8% (China), 7% (South Korea), and 6% (Sao Paulo, Brazil).^[10–14]^ Although multiple medications and surgical managements are available for CRNP, they are still ineffective at controlling its recurrence.^[15–19]^ Several studies found that nasal nebulization inhalation of budesonide (NNIB) can be used for CRNP treatment.^[20–22]^ However, the efficacy and safety of NNIB for the treatment of CRNP is still unclear at evidence-based medicine level. Therefore, this study aims to assess the efficacy and safety of NNIB for CRNP.

## 2 Methods and analysis

### 2.1 Study registration

This study has been registered on INPLASY202040108. It follows the guidelines of Preferred Reporting Items for Systematic review and Meta-Analysis Protocols.^[23,24]^

### 2.2 Eligibility criteria

#### 2.2.1 Type of studies

We will include relevant randomized controlled trials (RCTs) on investigating the efficacy and safety of NNIB for the treatment of CRNP. We will exclude any other studies, such as animal studies, reviews, case studies, non-controlled studies, and quasi-RCTs.

#### 2.2.2 Type of participants

All patients who were diagnosed as NNIB will be included regardless their race, age, and gender.

#### 2.2.3 Type of interventions

All patients in the experiment group received CRNP alone. We will exclude combination of CRNP with other interventions.

All participants in the control group undertook any management, but not the CRNP.

#### 2.2.4 Type of outcomes

The primary outcomes are nasal symptoms and polyp sizes. Nasal symptoms are assessed using Visual analogue scales, or other relevant scales. Polyp sizes are measured using Kennedy scores or other associated scores.

The secondary outcomes are serum cortisol levels, health-related quality of life (as checked using 36-Item Short Form Survey or other tools), and any expected and unexpected adverse events.

### 2.3 Search strategy

Studies will be identified by searching the following electronic databases from inception to the present: Cochrane Library, PubMed, EMBASE, Web of Science, PsycINFO, Cumulative Index to Nursing and Allied Health Literature, Allied and Complementary Medicine Database, Chinese Biomedical Literature Database, and China National Knowledge Infrastructure. No restrictions of language and publication status will be imposed in this study. The search strategy sample of PUBMED is exerted in Table 1. We will also adapt similar search strategies to other electronic databases.

**Table 1:**
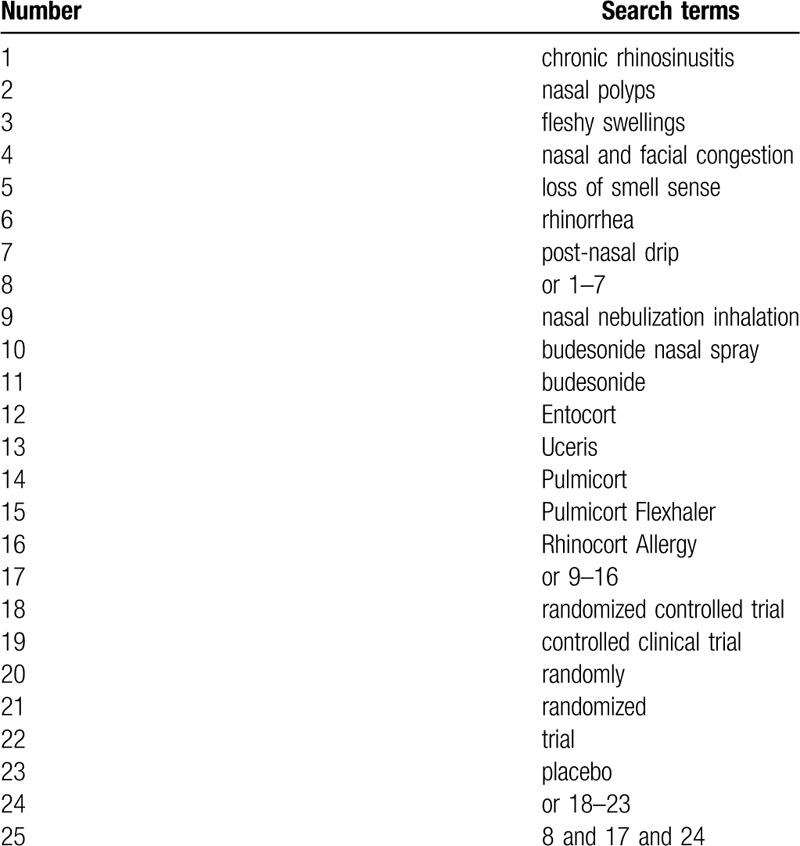
Search strategy of PubMed.

Additionally, this study will search any relevant conference proceedings, dissertations, sources of clinical trial registry, and reference lists of associated reviews.

### 2.4 Data collection and management

All identified records will be downloaded and managed in Endnote 7.0, and all duplicates will be excluded.

#### 2.4.1 Study selection

Two reviewers will independently screen titles and abstracts for all searched records, and all irrelevant studies will be removed based on the pre-specified eligible criteria. Any articles which appear eligible from the selection of abstracts, or are of unclear of eligibility, full-texts will be obtained to further check if they meet final eligibility criteria. Any disagreements between 2 reviewers over study selection will be discussed, and a third reviewer will be consulted to reach a consensus. We will note the reasons for all removed studies. In addition, we will show the whole process of study selection in a flow diagram.

#### 2.4.2 Data collection

After study selection, all important data will be collected using a data collection sheet, which will be piloted on the eligible studies before being finalized by 2 independent reviewers. Any disagreements between 2 reviewers over the data collection will be discussed, and a third reviewer will be consulted to reach a consensus. If there are multiple studies which utilized the same data sources, they will be noted separately in data collection as they may provide views to different covariates, and mediators. The following information will be collected: study characteristics (such as title, first author, year of publication, country, etc), patient characteristics (such as race, age, gender, diagnostic criteria, inclusion, and exclusion criteria, etc), study setting, sample size, study methods (such as details of randomization, blind, concealment, etc), details of interventions, and controls (such as types of interventions, controls, dosage, duration, etc), relevant outcome measurements, any expected and unexpected adverse event, and funding information.

### 2.5 Study quality assessment

All included studies will be evaluated using Cochrane risk of bias tool for RCTs. It is recommended by the Cochrane Handbook. It covers 7 aspects and each 1 is further graded as low risk of bias, unclear risk of bias or high risk of bias. All study quality assessment will be independently performed by 2 reviewers. Any divergences between 2 reviewers will be solved by a third reviewer through discussion to reach a final decision.

### 2.6 Data synthesis and analysis

#### 2.6.1 Data synthesis

RevMan 5.3 Software will be applied for statistical analysis in this study. All continuous values will be exerted as mean difference or standardized mean difference and 95% confidence intervals, while all dichotomous values will be presented as risk ratio and 95% confidence intervals. The heterogeneity among included studies will be identified using I^2^ statistic test and will be interpreted as follows: I^2^ ≤ 50% means low level of heterogeneity, and a fixed-effects model will be utilized to pool the data; while I^2^ > 50% indicates high level of heterogeneity, and a random-effects model will be used to synthesize the data. If low level of heterogeneity among included is identified, we will perform a meta-analysis if necessary. On the other hand, if there is a high degree of heterogeneity among primary RCTs, including different characteristics of study or patient, study designs, interventions, and controls, which will limit our ability to carry out a meta-analysis. Then, we will conduct a subgroup analysis to investigate possible reasons that may result in such obvious heterogeneity.

#### 2.6.2 Subgroup analysis

We will conduct subgroup analysis according to the different characteristics of study or patient, different interventions or controls, and different outcome measurements.

#### 2.6.3 Sensitivity analysis

If necessary, we will perform sensitivity analysis to check the robustness of pooled outcome results by removing low quality studies.

#### 2.6.4 Reporting bias

If at least 10 eligible RCTs are included, we will undertake funnel plot and Egger regression test to check if there is any possible reporting bias.^[25]^

### 2.7 Quality of evidence

All quality of evidence of each outcome will be identified using Grading of Recommendations Assessment Development and Evaluation.^[26]^ It will be assessed through 5 fields, and each 1 is further graded as high, moderate, low, or very low according to Grading of Recommendations Assessment Development and Evaluation rating standards.

### 2.8 Ethics and dissemination

No ethical approval is sought, because all individual data is not obtained in this study. This study is expected to be published on a peer-reviewed journal.

### 2.9 Patient and public involvement

Patients and/or the public were not directly involved in the development of the protocol of this study.

## 3 Discussion

Numerous clinical studies have reported the efficacy and safety of NNIB for the treatment of CRNP.^[20–22]^ It will comprehensively retrieve as more as possible electronic databases and other literature resources to identify potential studies. Two reviewers will independently undertake study investigation, data collection, and study quality evaluation. Any different views will be resolved by a third reviewer through discussion or consultation. The results of the present study may provide evidence to determine whether NNIB is effective and safety for the treatment of CRNP.

## Author contributions

**Conceptualization:** Peng-ju Zheng, Yi-ying Zhang, Shu-hua Zhang, Jin-sheng Wang.

**Data curation:** Peng-ju Zheng, Shu-hua Zhang, Jin-sheng Wang.

**Formal analysis:** Peng-ju Zheng, Yi-ying Zhang, Gui-fang Liu.

**Investigation:** Jin-sheng Wang.

**Methodology:** Peng-ju Zheng, Yi-ying Zhang, Shu-hua Zhang, Gui-fang Liu.

**Project administration:** Jin-sheng Wang.

**Resources:** Peng-ju Zheng, Yi-ying Zhang, Gui-fang Liu.

**Software:** Peng-ju Zheng, Yi-ying Zhang.

**Supervision:** Yi-ying Zhang, Jin-sheng Wang.

**Validation:** Peng-ju Zheng, Yi-ying Zhang, Shu-hua Zhang, Gui-fang Liu, Jin-sheng Wang.

**Visualization:** Peng-ju Zheng, Jin-sheng Wang.

**Writing – original draft:** Peng-ju Zheng, Yi-ying Zhang, Shu-hua Zhang, Gui-fang Liu, Jin-sheng Wang.

**Writing – review & editing:** Peng-ju Zheng, Yi-ying Zhang, Gui-fang Liu, Jin-sheng Wang.
